# Structural and Thermoelectric Properties of Gd_2−2*x*_Sr_1+2*x*_Mn_2_O_7_ Double-Layered Manganites

**DOI:** 10.3390/ma16072548

**Published:** 2023-03-23

**Authors:** Nailing Qin, Yehai Pang, Zhengbing Xu, Xiyong Chen, Jialin Yan

**Affiliations:** 1 School of Resources, Environment and Materials, Guangxi University, Nanning 530004, China; 2 State Key Laboratory of Featured Metal Materials and Life-Cycle Safety for Composite Structures, Nanning 530004, China

**Keywords:** double-layered manganites, crystal structure, thermoelectric properties, *n*-type perovskite oxides

## Abstract

Double-layered manganites are natural superlattices with low thermal conductivity, which is of importance for potential thermoelectric applications. The Gd_2−2*x*_Sr_1+2*x*_Mn_2_O_7_ (*x* = 0.5; 0.625; 0.75) were prepared by the solid-state reaction method. All the samples crystallize in the tetragonal I4/mmm Sr_3_Ti_2_O_7_ type structure. The unit cell volume and the distortion in the MnO_6_ octahedra increase with increasing Gd content. Their thermoelectric properties were investigated between 300 and 1200 K. All exhibit an *n*-type semiconducting behavior. The electrical conductivity (*σ*) increases while the absolute value of the Seebeck coefficient (|*S*|) decreases with increasing Gd content. Simultaneous increases in *σ* and |*S*| with increasing temperature are observed at temperatures approximately higher than 600 K, and the power factor reaches a maximum value of 18.36 μW/(m K²) for *x* = 0.75 at 1200 K. The thermal conductivity (*κ*) is lower than 2 W/(m K) over the temperature range of 300–1000 K for all the samples and a maximum dimensionless figure of merit *ZT* of 0.01 is obtained for *x* = 0.75 at 1000 K.

## 1. Introduction

Since the report of the layered NaCo_2_O_4_ showing good thermoelectric (TE) performance with a large Seebeck coefficient of 100 μV/K at 300 K [1], there has been increasing interest in exploring new oxide TE materials in the last two decades because of their high chemical and thermal stability at high temperature, low toxicity, and relatively low-cost starting materials [2,3]. TE materials enable the direct conversion of thermal into electricity and are useful for manufacturing TE devices for power generation from waste heat. The efficiency of a TE material is mainly determined by the dimensionless figure of merit *ZT*, which is a product of the TE figure of merit *Z* and the absolute temperature *T*, given by *ZT* = *S*^2^*σT/κ*, where *S*, *σ*, *κ*, and *T* are the Seebeck coefficient, electrical conductivity, thermal conductivity, and absolute temperature, respectively. A good TE material requires large *S*, high *σ*, and low *κ*. As these three parameters are strongly coupled, depending on the carrier concentration and electronic structure, and there is a trade-off between *S* and *σ*, it is difficult to enhance them simultaneously. The power factor *PF*, defined as *S*^2^*σ*, is related to the electrical properties of a material. Whilst the performance of most TE oxides is limited by their low *ZT* values due to low *PF* and high *κ*, several layer-structured oxides show outstanding TE properties, such as *p*-type layered cobaltites Ca_3_Co_4_O_9_ and BiCuSeO, and *n*-type perovskite oxides CaMnO_3_ manganates and SrTiO_3_ titanates [2,3,4]. Methods to enhance *ZT* mostly include doping, carrier engineering, defect chemistry engineering, nanostructuring/nanocomposites, band engineering, etc., aiming to improve *PF* and reduce *κ* [2,3,5,6,7].

The Ruddlesden–Popper (RP) compounds with a general formula A*_m_*_+1_B*_m_*O_3*m*+1_ (A = rare earth (RE) and/or alkaline earth elements and B = transition metals) or AO(ABO_3_)*_m_* have a natural superlattice structure consisting of an alternate stacking of multiple (*m*) perovskite structure BO_2_ layers and a single rock-salt A_2_O_2_ layer along the *c* axis direction [8]. The double-layered oxides A_3_B_2_O_7_ belong to the *m* = 2 member and the perovskites ABO_3_ correspond to the *m* = ∞ member of the RP family. The layered structure of the RP compounds would enhance the phonon scattering at the interfaces between the A_2_O_2_ layer and perovskite layer and consequently reduce *κ*, which is of great importance for TE materials. Investigation on thermal conductivity in the thin films of the *m* = 1–5 and 10 members of the (SrTiO_3_)*_m_*SrO RP superlattices showed that the *m* = 2 member had the lowest *κ* in this RP homologous series [9]. Significant reduction of *κ* values was observed in the layered (Sr_1−*x*_RE*_x_*)*_m_*_+1_Ti*_m_*O_3*m*+1_ (*m* = 1, 2) [10] and La_2−2*x*_Ca_1+2*x*_Mn_2_O_7_ (0.75 ≤ *x* ≤ 1.0) [11] due to the intrinsic superlattice structure as compared to their perovskite phases. It is desired for a TE material that good electron transport properties *PF* would be kept while *κ* is reduced. The structure of the RP oxides allows compositional tailoring, and the TE properties of *n*-type CaMnO_3_ and SrTiO_3_ can be improved by substitution at either the A or B sites. It has been found that RE element doping at Ca sites of CaMnO_3_ is an effective way to increase *σ* while keeping a moderate absolute *S* [6,12]. Studies of the effects of various RE^3+^ ions doping at Sr sites of (Sr_0.95_RE_0.05_)_3_Ti_2_O_7_ oxides on their TE properties indicated that the maximum *ZT* was obtained in Gd-doped (Sr_0.95_Gd_0.05_)_3_Ti_2_O_7_ mainly owing to its lower *κ* [13] or enhanced *S* [14].

The double-layered manganites RE_2−2*x*_Sr_1+2*x*_Mn_2_O_7_ are of significant interest due to the effect of colossal magnetoresistance (CMR) and intensive studies have been focused on their magnetic and magneto-transport properties [15,16,17,18,19,20,21]. However, works on the high-temperature TE properties of RE_2−2*x*_Sr_1+2*x*_Mn_2_O_7_ to take advantage of their intrinsic superlattice structures are very scarce. During the course of our systematic research on the phase diagram of the Gd–Sr–Co/Mn–O systems [22,23], a Gd_2−2*x*_Sr_1+2*x*_Mn_2_O_7_ solid solution was found. Our magnetic measurements of the Gd_2−2*x*_Sr_1+2*x*_Mn_2_O_7_ samples in the temperature range of 2–350 K under an applied magnetic field of 0.02 T show two ferromagnetic transitions, which is in analogy to the temperature-dependent magnetizations of La_1.2_Sr_1.8_Mn_2_O_7_ [24] and La_1.4_Sr_1.6_Mn_2_O_7_ [25]. In this paper, we report the structural and TE properties of double-layered manganites Gd_2−2*x*_Sr_1+2*x*_Mn_2_O_7_ (*x* = 0.5, 0.625, 0.75). Investigation on their TE properties revealed an *n*-type semiconducting behavior and a *κ* of lower than 2 W/(m K) over the temperature range of 300–1000 K. *σ* increases while the absolute value of *S* decreases with increasing Gd content.

## 2. Materials and Methods

Polycrystalline samples of Gd_2−2*x*_Sr_1+2*x*_Mn_2_O_7_ (*x* = 0.5, 0.625, 0.75) were prepared by the conventional solid-state reaction method in air. Gd_2_O_3_ (99.95%, Sinopharm, Beijing, China), SrCO_3_ (≥99.0%, Sinopharm, Beijing, China), and MnCO_3_ (99.95%, Aladdin, Shanghai, China) were used as starting materials. Gd_2_O_3_ and SrCO_3_ were dried at 773 K for 24 h, and MnCO_3_ at 373 K for 24 h prior to use. Stoichiometric amounts of the preheated powders were thoroughly mixed and ball-milled in anhydrous ethanol medium for 10 h in the agate grinding jars using a planetary ball mill (QM−3SP4, Nanjing, China). The resultant powders were calcined at 1123 K for 24 h in a muffle furnace. Subsequently, the calcined powders were reground, pressed into pellets with diameters of 15 mm and a thickness of ~4 mm or pressed into 20 mm long rectangular samples with widths of 4 mm and a thickness of ~3 mm, and sintered at 1673 K for 120 h.

Powder X-ray diffraction (XRD) data were collected on an x-ray diffractometer (Rigaku D/Max 2500V, Tokyo, Japan) using Cu *Kα* radiation over the angular range of 10° to 110° 2*θ*, with a step size of 0.02°. The XRD data were analyzed with the Rietveld method using the Fullprof program [26]. The microstructure of the sintered samples was examined by a field emission scanning electron microscope (FE-SEM, Hitachi SU8020, Tokyo, Japan) using the secondary electron (SE) mode. The chemical compositions of the samples were determined by the equipped energy-dispersive X-ray spectrometer (EDS, Oxford X-MAX80, Oxford, UK). Specimens for the measurements of *σ* and *S* were prepared by grinding and polishing the sintered rectangular samples into a typical dimension of 3.9 mm × 2.0 mm × 19.0 mm. All the surfaces of the specimens were carefully polished with SiC emery papers before measurement to ensure parallel ends. The temperature dependences of *σ* and *S* were measured on a multifunctional thermoelectric materials measurement system (Advance Riko ZEM-3M10, Yokohama, Japan) in the temperature range of 300–1200 K in a helium atmosphere. The specimen was placed vertically between the upper and lower Pt block electrodes in the infrared heating furnace, and two probes of the thermocouple were adjusted to attach to the longitudinal side of the specimen. *V*-*I* plot measurement was carried out to check whether the specimen was well contacted with the probes before the simultaneous measurements of *σ* and *S.* The thermal conductivity *κ* was calculated using the relationship *κ = DC_p_d*, where *D* is the thermal diffusivity, *C_P_* is the specific heat capacity and *d* is the bulk density. *D* was measured using the laser flash method (NETZSCH LFA 457, Selb, Germany) on disc specimens with diameters of 12.7 mm and a typical thickness of 1.5 mm. *C_P_* was measured using differential scanning calorimetry (NETZSCH STA 449 F3, Selb, Germany) under an argon atmosphere up to 1000 K. The bulk density *d* of the sintered discs was determined by Archimedes’ method (Shimadzu AUW220D, Kyoto, Japan).

## 3. Results and Discussion

### 3.1. Structural and Morphological Analysis

**Figure 1:**
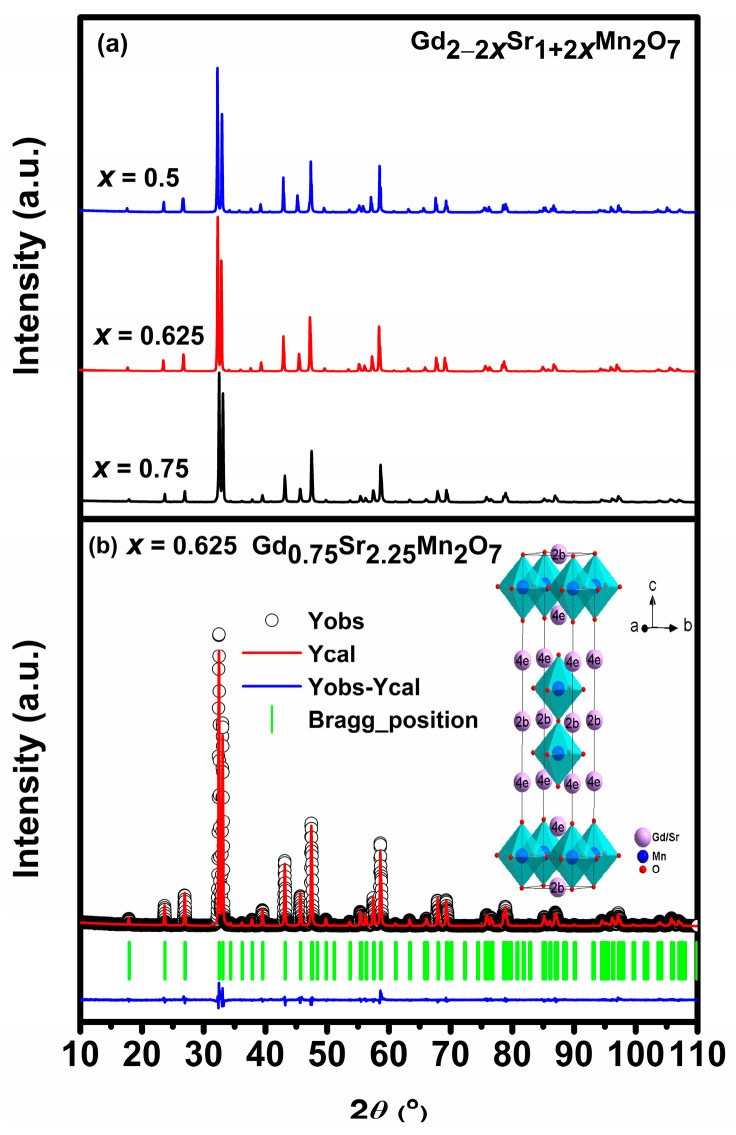
(**a**) Powder XRD patterns for Gd_2−2*x*_Sr_1+2*x*_Mn_2_O_7_ (*x* = 0.5, 0.625, 0.75). (**b**) Rietveld refinement pattern for *x* = 0.625. The inset shows the crystal structure of the unit cell.

Figure 1a shows the powder XRD patterns of Gd_2−2*x*_Sr_1+2*x*_Mn_2_O_7_ (*x* = 0.5, 0.625, 0.75). All the diffraction peaks can be indexed in a tetragonal Sr_3_Ti_2_O_7_ type structure with space group I4/mmm (No. 139). A typical Rietveld refinement pattern and the crystal structure for the sample *x* = 0.625 are demonstrated in Figure 1b. As seen from the structure, two stacked MnO_2_ layers (i.e., double perovskite layers) form the quasi-two-dimensional (2D) magnetic layer (called bilayer). Two adjacent MnO_2_ bilayers are separated by the (Gd, Sr)_2_O_2_ rock salt layers. The refined structural parameters, theoretical density (*ρ_x_*), selected bond lengths, and reliability factors for Gd_2−2*x*_Sr_1+2*x*_Mn_2_O_7_ are summarized in Table 1. The measured bulk density d of the sintered samples and the relative density (% T. D.) are also given in Table 1. The relative density for all the samples is about 94%, indicating that the samples are of similar compactness.

According to the analysis from the neutron powder diffraction data [27], the Sr/RE ions occupy two Wyckoff sites, i.e., 2b (0, 0, 1/2) site in the 12-coordinate perovskite-like block and 4e (0, 0, z) site in the 9-coordinate rock salt layer. As seen in Figure 1b, each Gd^3+^/Sr^2+^ ion is labeled by 2b or 4e to indicate its atomic Wyckoff position. Smaller RE ions such as Gd^3+^, Tb^3+^, Dy^3+^, etc., were found to prefer the 4e site [27]. Refinements on the occupancies of Gd^3+^/Sr^2+^ ions showed that they co-occupied the 2b and 4e sites with a higher occupation of Gd^3+^ ions at the 4e sites. The refined occupancies of Gd^3+^/Sr^2+^ ions are given in Table 1. This is in agreement with the results of the refinement of DySr_2_Mn_2_O_7_ [27] and (Sr_0.95_RE_0.05_)_3_Ti_2_O_7_ [14]. This preferential occupation of RE^3+^ ions at the 4e sites might be due to the smaller ionic radius differences between RE^3+^ ions and 9-coordinate Sr^2+^ (r = 1.31Å, coordination number (CN) = 9) as compared to 12-coordinate Sr^2+^ (r = 1.44Å, CN = 12) [14]. With increasing Gd content, the unit cell volume increases while the unit cell parameter a first increases and then decreases, and c shows the opposite variation with a. These size variations have been found for Nd_0.2_La_1.8−2*x*_Sr_1+2*x*_Mn_2_O_7_ (0.3 *≤ x ≤* 0.7) [28], and can be attributed to the simultaneous occurrence of the substitution of Sr^2+^ (r = 1.31Å, CN = 9) ions with smaller Gd^3+^ ions (r = 1.107Å, CN = 9) and the conversion of Mn^4+^ (r = 0.53 Å, CN = 6) to larger Mn^3+^ (high spin, r = 0.645 Å, CN = 6) to maintain charge neutrality. It is therefore expected that the increasing amount of the Jahn–Teller active Mn^3+^ ions would lead to a stronger MnO_6_ octahedral distortion with increasing Gd content in Gd_2−2*x*_Sr_1+2*x*_Mn_2_O_7_. The tolerance factor t describes the structural distortion, defined as $t=\left(\langle r_{A}\rangle +r_{O}\right)/\left[\sqrt{2}\left(\langle r_{B}\rangle +r_{O}\right)\right]$, where $r_{O}$ is the ionic radius of the O ion and $\langle r_{A}\rangle$ and $\langle r_{B}\rangle$ are the mean radii of the ions at the A and B sites, respectively. With increasing Gd content, $\langle r_{A}\rangle$ decreases and $\langle r_{B}\rangle$ increases, a decreasing *t* is then obtained which confirms the enhancement of the structural distortion. Accordingly, the bond lengths of the apical Mn–O1 bonds (Mn to the apical oxygen atom O1 shared between the two MnO_2_ layers within a bilayer) and the in-plane Mn–O3 bonds (Mn to the equatorial oxygen atom O3 in the MnO_2_ layers) vary oppositely, with the Mn–O1 bonds along the c axis showing a larger extent of variation. The bond lengths of the apical Mn–O2 bonds (Mn to the apical oxygen atom O2 in the (Gd/Sr)_2_O_2_ rock-salt layers) are longer than those of the apical Mn–O1 and in-plane Mn–O3 bonds, and are elongated with increasing Gd content, which indicates the enlargement of the interlayer Mn–Mn distances.

**Figure 2:**
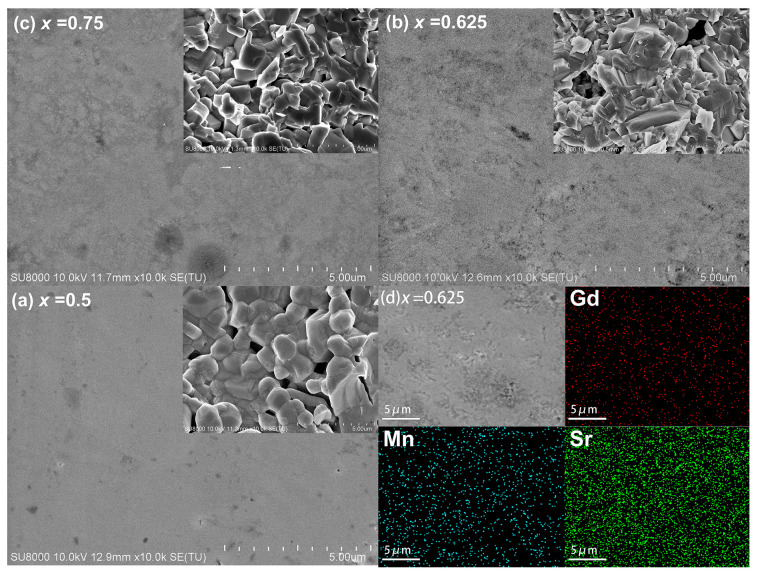
FE-SEM micrographs of the polished surface of sintered Gd_2−2*x*_Sr_1+2*x*_Mn_2_O_7_ with (**a**) *x* = 0.5, (**b**) *x* = 0.625, (**c**) *x* = 0.75. The insets of (**a**–**c**) are the fractured surface micrographs. (**d**) EDS mapping images for *x* = 0.625.

Figure 2a–c presents the FE-SEM polished surface micrographs of sintered pellets of Gd_2−2*x*_Sr_1+2*x*_Mn_2_O_7_ (*x* = 0.5, 0.625, 0.75) samples. The insets of Figure 2a–c are the fractured surface micrographs. Samples *x* = 0.625 and *x* = 0.75 show similar polished surface morphologies, with an uneven surface and opened pores. It is evident from the insets that the fractured morphology evolves from slightly aggregated spherical shape grains for *x* = 0.5 to lath-like or even flake-shaped grains for *x* = 0.625 and *x* = 0.75, respectively. The fractured morphologies of all the samples show a dense structure, which is consistent with the relative density of 94% obtained from Archimedes’ method (Table 1). Figure 2d shows the EDS mapping images of the cations for *x* = 0.625, indicating homogeneous distributions of the elements Gd, Sr, and Mn. The compositions measured by EDS are given in Table 2. It is shown that the molar ratios of the cations correspond well to the nominal compositions.

### 3.2. Thermoelectric Properties

**Figure 3:**
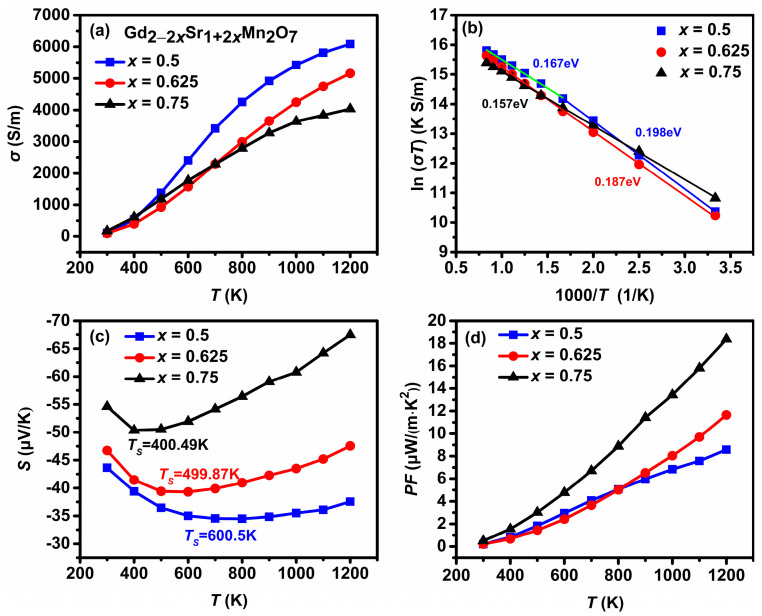
(**a**) Temperature dependence of the electrical conductivity, (**b**) plot of ln(*σT*) versus 1000/*T*. The solid lines represent the fitting of the small polaron hopping model. Temperature dependence of (**c**) the Seebeck coefficient, and (**d**) the power factor for Gd_2−2*x*_Sr_1+2*x*_Mn_2_O_7_ (*x* = 0.5, 0.625, 0.75).

Figure 3a shows the temperature dependence of *σ* for Gd_2−2*x*_Sr_1+2*x*_Mn_2_O_7_ (*x* = 0.5, 0.625, 0.75) in the temperature range of 300–1200 K. All the samples exhibit a semiconducting behavior with *dσ/dT >* 0. *σ* increases gradually with increasing Gd content at the high-temperature region, and a maximum value of 6.1 × 10^3^ S/m at 1200 K is observed for *x* = 0.5, which is of the same order of magnitude as those for Ca_0.96_Dy_0.02_RE_0.02_MnO_3_ [29]. As the substitution of Gd^3+^ ions for Sr^2+^ ions induces Mn^3+^ ions and donates electrons, where the nominal amount of Mn^3+^ ions (i.e., nominal electron concentration) can be estimated from the composition subscript (2−2*x*) while the nominal amount of Mn^4+^ ions (nominal hole concentration) estimated from the composition subscript (2*x*), the Jahn–Teller distortion of the Mn^3+^ ions leads to the formation of polarons where the electrons are localized due to the strong electron–phonon coupling. The electrical transport of Gd_2−2*x*_Sr_1+2*x*_Mn_2_O_7_ is thought to be dominated by the hopping motions of electrons or small polaron between Mn^3+^ and Mn^4+^ ions. The small polaron hopping conduction can be expressed by the equation [30]:

(1) $$\sigma \left(T\right)=\frac{\sigma_{0}}{T}exp\left(-\frac{E_{a}}{k_{B}T}\right)$$

where $\sigma_{0}$ is the pre-exponential constant, *E_a_* is the activation energy of small polaron hopping, *k*_B_ is Boltzmann constant, and *T* is the absolute temperature. The values of *E_a_* were deduced from the slope of the plot of ln (*σT*) versus 1000/*T*. As shown in Figure 3b, good linear fittings were obtained over the whole temperature range for *x* = 0.625 and 0.75, and *E_a_* was found to be 0.187 and 0.157 eV, respectively. A change in slope was observed for *x* = 0.5, with an *E_a_* of 0.198 eV at the 300–600 K region and 0.167 eV at the 600–1200 K region. It is found that *E_a_* increases with Gd content below 600 K, that is, 0.198, 0.187, and 0.157 eV for *x* = 0.5, 0.625, and 0.75, respectively. This may be attributed to the increasing concentration of Mn^3+^ Jahn–Teller ions which is favorable for the formation of small polarons in this temperature range [31].

The values of *S* are negative in the whole measured temperature range as seen in Figure 3c, indicating that electrons are the dominant charge carriers. *σ* increases while the absolute value of *S* decreases with increasing Gd content due to the increase in electron carrier concentration, which is similar to those for La_2−2*x*_Ca_1+2*x*_Mn_2_O_7_ (0.75 ≤ *x* ≤ 1.0) [11], La_2−2*x*_Sr_1+2*x*_Mn_2_O_7_ [32], and Gd_1−*x*_Sr*_x_*MnO_3_ (*x* = 0.5, 0.6, 0.7, 0.8) [33]. The absolute values |*S*| at 1200 K are found to be decreased by 44% from 67.5 μV/K for *x* = 0.75 to 37.5 μV/K for *x* = 0.5. It is observed that the absolute values |*S*| initially decrease and then increase with temperature, showing a change from a typical semiconducting behavior to a metallic or degenerate semiconducting behavior, which does not coincide with the temperature dependence of *σ*. The mechanism behind this phenomenon is not clear at present. The temperature *Ts* which are marked in Figure 3c corresponding to the minimum |*S*| value, or the so-called metal–insulator transition temperature, was found to shift to higher temperature with increasing Gd content owing to higher electron carrier concentration. Above *Ts,* all the samples exhibit a metallic or degenerate semiconducting behavior arising from the enhanced scattering of electrons at high temperatures. The dependence of *S* on the carrier concentration *n* and temperature *T* for degenerate semiconductors can be expressed as [34]:

(2) $$S=\frac{8\pi^{2}k_{B}^{2}}{3eh^{2}}m^{*}T{\left(\frac{\pi }{3n}\right)}^{\frac{2}{3}}$$

where m* is the effective mass of the carrier, *k_B_* is the Boltzmann constant, *e* is the elementary charge, and *h* is Plank’s constant. It is seen from Figure 3a that as *T* increases from room temperature to 600 K, *σ* increases sharply from ca. 1.0 × 10^2^ S/m to 2.0 × 10^3^ S/m, implying a rapid increase in electron carrier concentration *n*. This rapid increasing *n* at low temperatures is the dominant factor for the initial decrease in |*S*| because |*S*| is inversely proportional to the electron concentration. Then |*S*| gradually increases with further increasing temperature for an approximately given carrier concentration. Simultaneous increases in *σ* and |*S*| with increasing temperature are observed above *Ts*. This phenomenon was also found in CaMnO_3−δ_ [12] and Yb_0.1_Ca_0.9_Mn_1−*x*_Nb*_x_*O_3_ (*x* = 0.08, 0.1) [35], and was explained by using a two-band model of *S* which consists of contributions from the hole (Mn^4+^) and electron (Mn^3+^) due to the existence of mixed-valence Mn^3+^ and Mn^4+^. This model may be applicable to the present Gd_2−2*x*_Sr_1+2*x*_Mn_2_O_7_. Figure 3d shows the temperature dependence of the power factor *PF*. The monotonic increase in *PF* with temperature for all the samples is obtained due to the increases in both *σ* and |*S*| at high temperatures. The *PF* increases with decreasing Gd content and a maximum value of 18.36 μW/(m K²) is observed for *x* = 0.75 at 1200 K.

**Figure 4:**
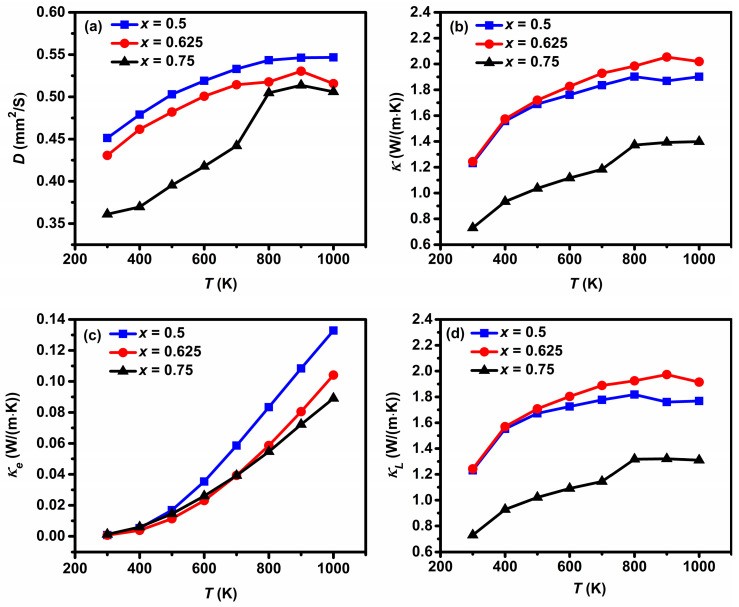
Temperature dependence of (**a**) the thermal diffusivity, (**b**) the measured thermal conductivity, (**c**) the electronic thermal conductivity, and (**d**) the lattice thermal conductivity for Gd_2−2*x*_Sr_1+2*x*_Mn_2_O_7_ (*x* = 0.5, 0.625, 0.75).

**Figure 5:**
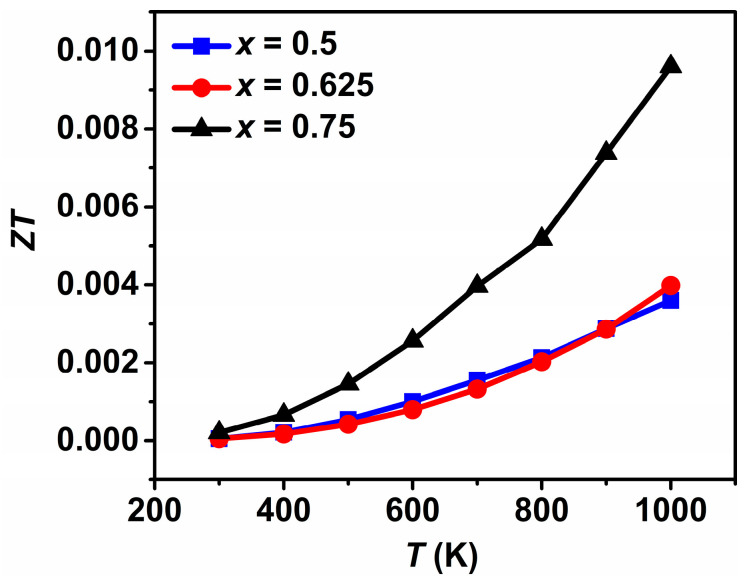
Temperature dependence of *ZT* for Gd_2−2*x*_Sr_1+2*x*_Mn_2_O_7_ (*x* = 0.5, 0.625, 0.75).

The measured *κ* was obtained using the relationship *κ = DC_p_d*. Measurements for *D* and *C_P_* were carried out from 300 to 1000 K. As seen from Figure 4a, *D* increases with increasing temperature and with Gd content. *C_P_* also increases with increasing temperature, ranging from 0.46, 0.51, 0.36 J/(g K) at 300 K to 0.58, 0.69, 0.50 J/(g K) at 1000 K for *x* = 0.5, 0.625, 0.75, respectively. The values of *D* and *C_P_* at 300 K are comparable to those for La_2−2*x*_Ca_1+2*x*_Mn_2_O_7_ in Ref. [11]. Figure 4b shows the temperature dependence of measured *κ* for Gd_2−2*x*_Sr_1+2*x*_Mn_2_O_7_ (*x* = 0.5, 0.625, 0.75). *κ* is lower than 2 W/(m K) over the measured temperature range. *κ* increases slightly with temperature and with increasing Gd content, which is constant with those for La_2−2*x*_Ca_1+2*x*_Mn_2_O_7_ in Ref. [11]. The measured *κ* consists of two contributions from phonons and electron carriers, i.e., *κ = κ_L_ + κ_e_*, the lattice thermal conductivity *κ_L_*, and the electronic thermal conductivity *κ_e_*. *κ_e_* can be calculated according to the Wiedemann–Franz law, *κ_e_ = LσT*, where *L* is the Lorentz number (2.45 × 10^−8^ W Ω/K^2^). Increasing *σ* is accompanied by an increase in *κ_e_*. Temperature-dependent *κ_e_* and *κ_L_* are presented in Figure 4c,d. The values of *κ_e_* are seen to be one order of magnitude smaller than those of *κ_L_*, indicating that a significant contribution is related to the lattice vibration for the heat transport in Gd_2−2*x*_Sr_1+2*x*_Mn_2_O_7_. Figure 5 shows the temperature dependence of *ZT*. A similar trend is observed for *ZT* and *PF*. The *x* = 0.75 compound Gd_0.5_Sr_2.5_Mn_2_O_7_ shows better performances than the other ones due to the simultaneously enhanced *S* and reduced *κ*, with a *σ* of 3.6 × 10^3^ S/m, a *S* of −60.8 μV/K, and a *κ* of 1.4 W/(m K) at 1000 K. A maximum *ZT* of 0.01 is thus obtained for *x* = 0.75, which is comparable to that of *n*-type La_2−2*x*_Ca_1+2*x*_Mn_2_O_7_ (0.75 ≤ *x* ≤ 1.0) [11] and *p*-type Ca_3_Co_2−*x*_Mn*_x_*O_6_ [36]. The *ZT* value is very low for practical TE applications. These results reveal that efforts should be made to enhance *σ* and/or *S* through compositional and processing optimizations in order to obtain high *ZT* for these double layered manganites to be used as potential candidates for *n*-type TE materials.

## 4. Conclusions

The structure and TE properties of Mn^3+^/Mn^4+^ mixed-valence double-layered manganites Gd_2−2*x*_Sr_1+2*x*_Mn_2_O_7_ (*x* = 0.5, 0.625, 0.75) were studied. XRD patterns of the samples were consistent with a tetragonal Sr_3_Ti_2_O_7_ type structure with space group I4/mmm (No. 139). The Rietveld refinements indicated that the unit cell volume and the distortion in the MnO_6_ octahedra increase with increasing Gd content. SEM micrographs and EDS measurements of all the samples show dense and uniform microstructures. All the samples are *n*-type semiconductors, and *σ* can be fitted well by the small polaron hopping model in the whole temperature range. With increasing Gd content, *σ* increases while |*S*| decreases due to the increasing electron carrier concentration. It is found that the absolute values |*S*| initially decrease and then increase with temperature, and both *σ* and |*S*| increase with temperature approximately above 600 K, resulting in a monotonic increase in *PF*. These phenomena are interesting, and the physical mechanisms are worthy of further study. *κ* increases with increasing Gd content, and is lower than 2 W/(m K) over the temperature range of 300–1000 K. A maximum *ZT* of 0.01 is obtained for *x* = 0.75 at 1000 K. Although the *ZT* of Gd_2−2*x*_Sr_1+2*x*_Mn_2_O_7_ does not yet reach the performance required for practical TE materials, the present study sheds light on their electrical and thermal transport properties and the relevant mechanisms and lays a foundation for seeking new applications for these double-layered manganites.

## Figures and Tables

**Table 1:** Structural parameters obtained from Rietveld refinements for Gd_2−2*x*_Sr_1+2*x*_Mn_2_O_7_. Space group I4/mmm (No. 139), atomic Wyckoff positions: Gd1/Sr1, 2*b* (0, 0, 1/2), Gd2/Sr2, 4*e* (0, 0, *z*), Mn, 4*e* (0, 0, *z*), O1, 2*a* (0, 0, 0), O2, 4*e* (0, 0, *z*), O3, 8g (0, 1/2, *z*).

	*x* = 0.5	*x* = 0.625	*x* = 0.75
*a* (Å)	3.82705 (5)	3.83642 (7)	3.83446 (10)
*c* (Å)	20.0030 (3)	19.8882 (4)	19.9048 (6)
*V* (Å^3^)	292.971 (7)	292.72 (1)	292.66 (1)
*ρ_x_* (g/cm^3^) *	6.342	6.123	5.842
*d* (g/cm^3^)	5.9716	5.6942	5.5009
*% T. D.*	94.2	93.0	94.2
*Occ. Gd1/Sr1*	0.141/0.859	0.176/0.824	0.131/0.869
*z_Gd2/Sr2_*	0.31729 (4)	0.31714 (4)	0.31677 (5)
*Occ. Gd2/Sr2*	0.466/0.534	0.306/0.694	0.150/0.850
*z_Mn_*	0.09766 (8)	0.09698 (9)	0.0975 (1)
*z_O2_*	0.1996 (3)	0.1973 (3)	0.1966 (4)
*z_O3_*	0.0989 (2)	0.0969 (2)	0.0959 (3)
*d_Mn-O1_* (Å)	1.9535 (18)	1.9288 (18)	1.9417 (24)
*d_Mn-O2_* (Å)	2.0391 (63)	1.9952 (62)	1.9716 (83)
*d_Mn-O3_* (Å)	1.9137 (1)	1.91821 (4)	1.9175 (1)
*Rp* (%)	10.9	10.7	12.8
*Rwp* (%)	12.7	12.7	15.2
*Rexp* (%)	7.38	7.09	6.69

* Calculated using the relationship $\rho_{x}=ZM/\left(N_{A}V\right)$, where *Z* is the number of formula units per unit cell, *M* is the sum of the atomic weights of all cations and all anions in the formula unit, $N_{A}$ is Avogadro’s number and *V* is the volume of the unit cell. Here *Z* = 2. *M* was calculated from the compositions obtained from the refined occupancies, not from the nominal compositions.

**Table 2:** The measured compositions from EDS results for Gd_2−2*x*_Sr_1+2*x*_Mn_2_O_7_ (*x* = 0.5, 0.625, 0.75).

		EDS Composition (at.%)			
*x*	Nominal Formula	Gd	Sr	Mn	EDS Formula
0.5	GdSr_2_Mn_2_O_7_	20.77 ± 3.53	40.02 ± 1.48	39.21 ± 3.28	Gd_1.038_Sr_2.001_Mn_1.961_O_7_
0.625	Gd_0.75_Sr_2.25_Mn_2_O_7_	14.07 ± 4.12	46.25 ± 4.28	39.68 ± 1.92	Gd_0.703_Sr_2.313_Mn_1.984_O_7_
0.75	Gd_0.5_Sr_2.5_Mn_2_O_7_	12.64 ± 3.79	46.49 ± 3.67	40.87 ± 1.67	Gd_0.632_Sr_2.325_Mn_2.043_O_7_

## Data Availability

Data are contained within the article.
